# Impacts of tilapia aquaculture on native fish diversity at an ecologically important reservoir

**DOI:** 10.7717/peerj.15986

**Published:** 2023-12-19

**Authors:** Muzzalifah Abd Hamid, Amir Shah Ruddin Md Sah, Izwandy Idris, Siti Azizah Mohd Nor, Mashhor Mansor

**Affiliations:** 1 South China Sea Repository and Reference Centre, Institute of Oceanography and Environment, Universiti Malaysia Terengganu, Kuala Nerus, Terengganu, Malaysia; 2 School of Biological Sciences, Universiti Sains Malaysia, USM Minden, Pulau Pinang, Malaysia; 3 Institute of Climate Adaptation and Marine Biotechnology, Universiti Malaysia Terengganu, Kuala Nerus, Terengganu, Malaysia

**Keywords:** Tilapia, Aquaculture, Escapee, Temengor Reservoir, Catch per unit effort (CPUE), Native fish

## Abstract

**Background:**

The Temengor Reservoir is the second largest reservoir in Peninsular Malaysia. Located in the northwestern state of Perak, it was selected to develop a large-scale tilapia (*Oreochromis niloticus*) aquaculture facility within the Aquaculture Industrial Zone (AIZ) in 2008 due to its favourable environmental conditions. No record of tilapia has ever been reported in the natural waters prior to this. However, a post-establishment study recorded tilapia sightings in the natural waters of this lake. The cultured tilapia was easily recognizable with the elongated mouth and body, and long caudal fin. It is postulated that these were escapees from the floating cages that had invaded the natural waters and would negatively impact the native fish species. To test our hypothesis, we investigated the impact of the aquaculture facility on native fish diversity through a spatial design.

**Methods:**

The study was focused on assessing the impact of tilapia culture at sites nearer to the AIZ *vs* more distant sites, the former with a greater likelihood of receiving escapees. Two major sites were chosen; within 5 km (near-cage) and within 5–15 km (far-cage) radii from the AIZ. Fish sampling was conducted using multiple mesh sizes of gill nets (3.7, 5.1, 6.5, 7.6, and 10.2 cm) deployed at the littoral zone of the sampling points. Species diversity, abundance, dietary habits, and habitat preference were investigated.

**Results:**

The CPUE (individual/hour) of native fish species at the far-cage site of the AIZ Reservoir was found to be significantly higher (*p* < 0.05) than that at the near-cage site. Principal component analysis (PCA) based on diet and habitat preferences showed that the tilapia, *O. niloticus* had almost overlapping diet resources and habitat with native fish species.

**Conclusion:**

We conclude that there is a correlation between the reduced catches of native species (based on CPUE) and the high presence of tilapia. Thus, appropriate actions must be implemented for strategic and effective planning in terms of native fish conservation.

## Introduction

Aquaculture and fisheries are regarded as key drivers of the blue economy for sustainability and food security by most government and the private sectors in developing countries (Aura et al., 2021). Notably, Béné et al. (2016) highlighted that aquaculture had been increasingly viewed as a solution to the depletion of the world’s fisheries. Global aquaculture production expanded more than triple in live-weight volume from 34 MT in 1997 to 112 MT in 2017, with freshwater aquaculture significantly contributing to aquatic food supplies and nutrition security (Naylor et al., 2021).

In Malaysia, a large-scale aquaculture facility has been established at Temengor Reservoir, Perak, the second largest reservoir in Peninsular Malaysia. The aquaculture project was initiated in late 2008 within an area referred to as the Aquaculture Industrial Zone (AIZ), with a focus on the new strain of Genetically Improved Farmed Tilapia (GIFT) (Jamtøy, Ping & Alvarez, 2011; Hashim, 2015; Jumatli & Ismail, 2021; Abd Hamid et al., 2022a). The GIFT Foundation International Incorporated (GFII) entered into an agreement with GenoMar for dissemination rights of GIFT, which is rebranded as GenoMar Supreme Tilapia (GST). The project was a success, confirming the GIFT strain of *O. niloticus* as exhibiting high growth performance, high survival rates, high fillet weights, good flesh quality, resistance to disease, and well adapted to various farming systems (Ng & Hanim, 2007). However, on the downside, tilapia is also listed by the IUCN as one of the world’s top 100 aggressive invasive species (Lowe et al., 2000).

There was tilapia species recorded by Abd Hamid & Mansor (2013) in the natural waters of Temengor Reservoir. The presence of this species might be due to the “leakage” from the fish cages, as there was no tilapia recorded before 2009 (Haslawati, Raja Yana Maleesa & Mohd Nazri, 2021). Even though the tilapia at this lake is cultured in cages, there is always a risk that the fish could accidentally escape into the wild. This is because aquaculture is mainly responsible for introducing and establishing cultured species in local ecosystems through their escape into the wild (Casal, 2006). Since all captive tilapias (in the industry or research) could potentially escape, concerns have been raised over the negative impact of these invasions on native fish diversity (Peterson, Slack & Woodley, 2005; Senanan & Bart, 2010). Escaped tilapias could threaten native species through competition for food resources, niche displacement, and predation on native species (Kour, Bhatia & Sharma, 2014). Hence this could adversely affect the population sizes of native freshwater fishes (Britton, Gozlan & Copp, 2011; Beatty & Morgan, 2013).

Based on initial reports on tilapia sightings in the waters and their consequent immense threat to native fishes, it is vital to study the impact at Temengor Reservoir. The reservoir represents an ecologically important ecosystem in supporting as many as 42 species of freshwater fish, including the snakehead *Channa micropeltes*, bulu barb *Puntioplites bulu*, carp *Hampala macrolepidota*, and river catfish *Hemibagrus* spp. (Shah et al., 2016a). This lake is known to be the home of two endangered species, although the current status is not known; the mahseer *Tor tambroides* and Jullien’s golden carp *Probarbus julllieni* (Hashim et al., 2012). Therefore, while aquaculture at Temengor Reservoir holds great promise for economic growth and protein source, this study is necessary to evaluate the impact of tilapia aquaculture on native fish diversity and to plan a management strategy for protecting and conserving native fish communities in this man-made lake.

## Materials and Methods

### Fish sampling and assessment

**Figure 1:**
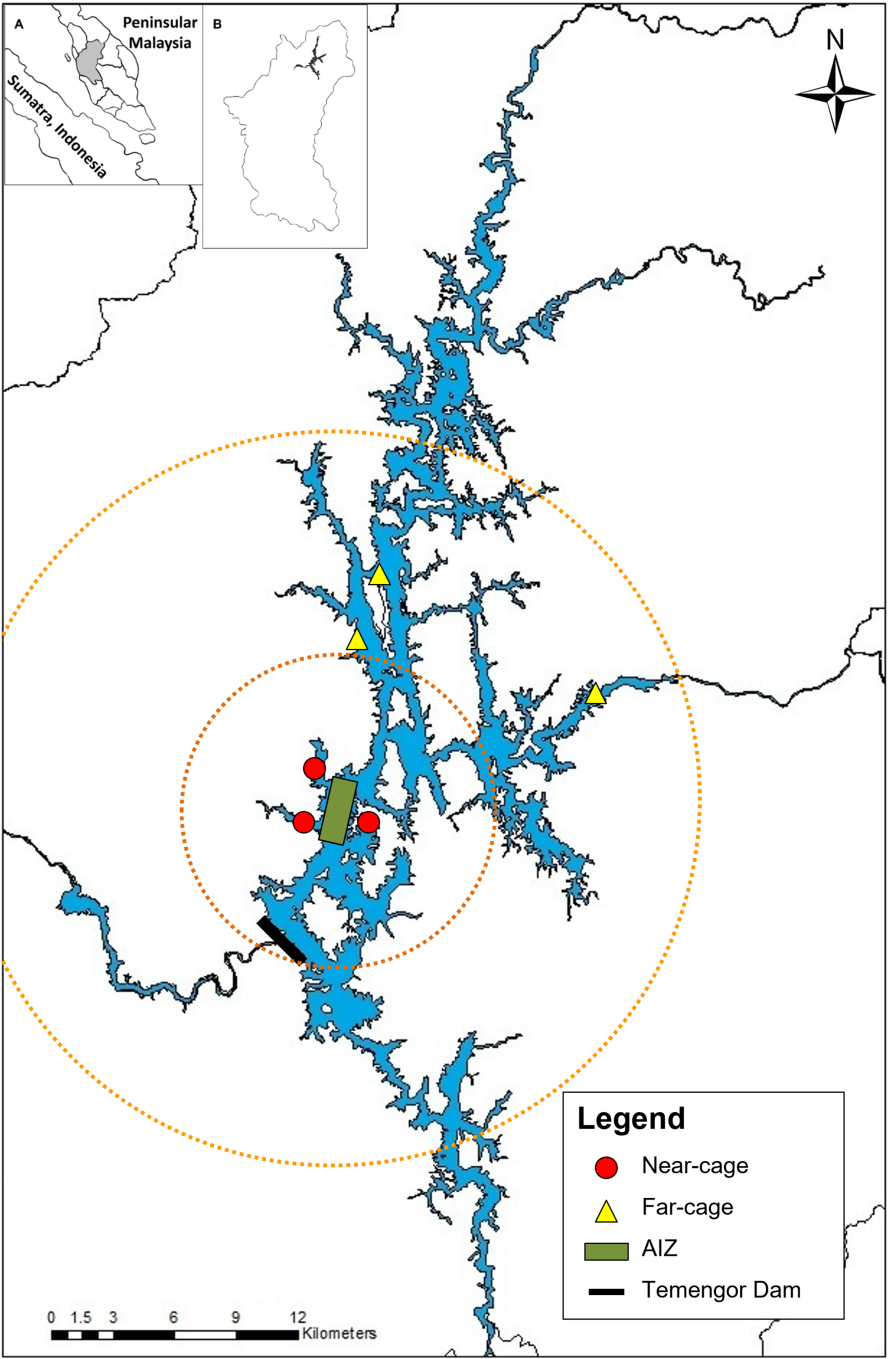
The sampling locations at near-cage and far-cage sites from the AIZ of Temengor Reservoir, Perak. Orange circle showing radius from AIZ; small circle denotes a 5 km radius (near-cage site) whereas larger circle denotes 5–15 km radius from AIZ (far-cage site). (Inset: (A) Sumatra and Peninsular Malaysia; (B) Perak state).

Monthly sampling was conducted from January 2014 to May 2015 at Temengor Reservoir at two major sites; near-cage (in the vicinity of the AIZ) and far-cage (distant from cage culture) (Fig. 1). The impacts of the tilapia fish aquaculture facilities were assessed by comparing the native fish assemblages between the spatial gradient. Arthur et al. (2010) stated that the impact of tilapia culture could be investigated through comparisons in the abundance or composition of the native fish community at impacted and non-impacted reference sites. Therefore, in this study, the far-cage site (non-impacted) were sampled from 5 to 15 km radius from the AIZ, while points within the 5 km radius were considered the “impacted” sites.

All fish specimens were collected with the help of local fishermen. During each sampling excursion, two sets of experimental gill nets (250 cm vertical length × 2,976 cm total width) comprising each of five different stretch mesh sizes (3.7, 5.1, 6.5, 7.6, 10.2 cm) were deployed randomly clustered (Brown & Austen, 1996) at three points at each near-cage site and far-cage site (Fig. 1) and left overnight (Hubert & Fabrizio, 2007; Shah et al., 2016b). The nets were deployed at the littoral zone of the lentic ecosystem of Temengor Reservoir, which is the most productive fish catch area due to its significance as a habitat for fish spawning, larval and juvenile development, or feeding (Werner et al., 1977; Schlosser, 1982; Ali, 1996; Ambak & Jalal, 2006). All sampling conditions were standardised, *i.e*., at both sites, six sets of gill nets (two sets × three points) were used and deployed at littoral zones from 5.30–6.30 pm, left overnight, and fish specimens were collected from 7.30–8.30 am.

**Figure 2:**
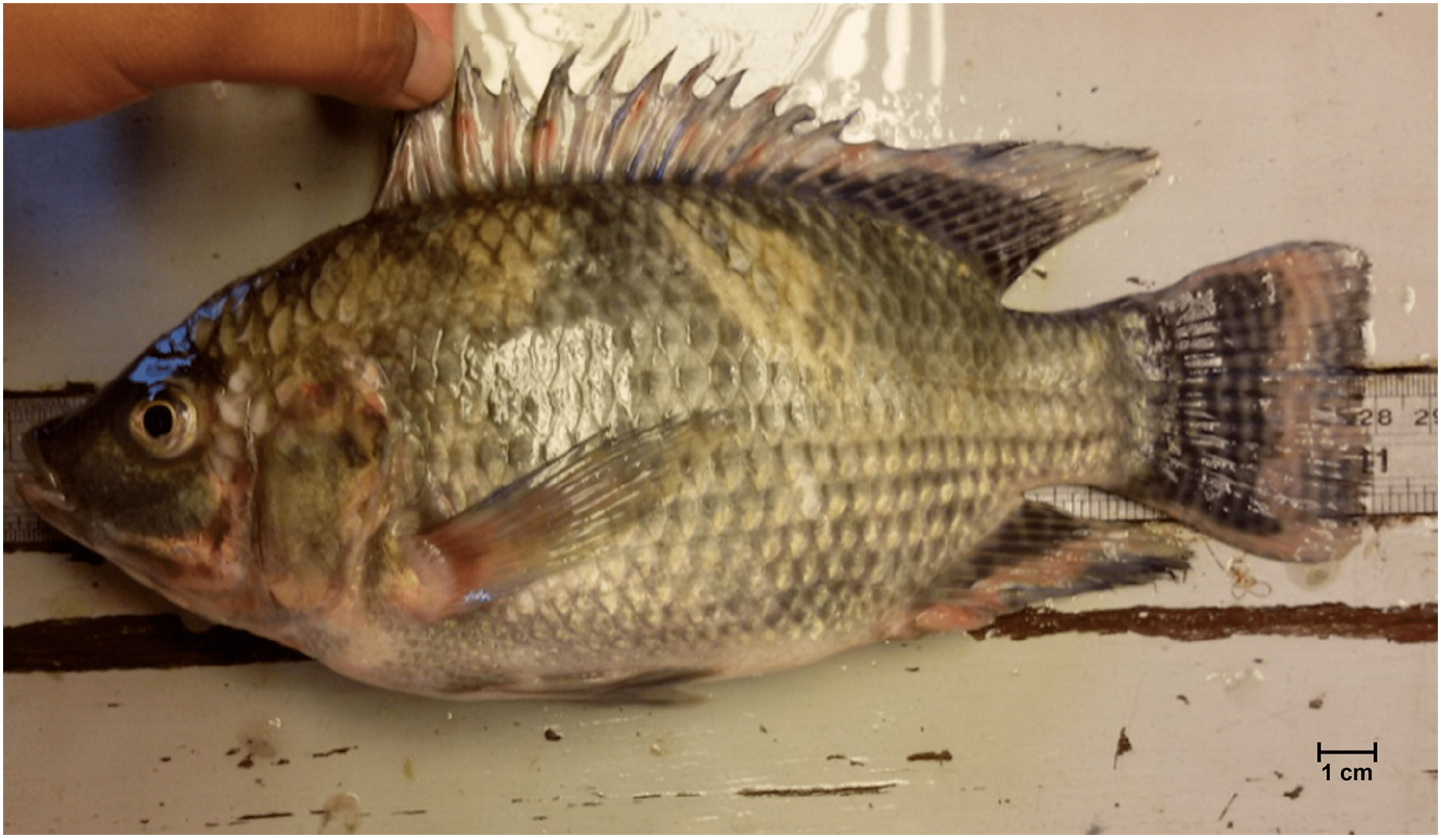
The cultured GenoMar Supreme Tilapia (GST), *Oreochromis niloticus* with elongated mouth and body, and long caudal fin captured in the natural waters of Temengor Reservoir.

All captured fish individuals were not euthanized and anesthetized as this study did not involve further experimental work after the taxonomic identification of each specimen. When caught, most specimens were already dead, and the few live samples did not survive more than a few hours. They were kept in an ice chest and taken back to the Pulau Banding Rainforest Research Centre for detailed identification. Species identification was based on the taxonomic keys by Ambak et al. (2010) and Kottelat (2013). The number of individuals per species was counted. Escaped GST tilapia (Fig. 2) was easily recognizable from its distinctive characteristics; elongated mouth and body, and long caudal fin (Mat-Taib SA., Farm manager of Trapia Malaysia Sdn. Bhd., 2016, personal communication).

### Data analyses

**Species frequency–**The species frequency was calculated based on Othman, Nor & Besar (2002). Species represented by less than 2% of the total catch were classified as “less frequent” whereas those constituting more than 2% were classified as “frequent”.

**Catch per unit effort (CPUE)–**All absolute values of abundances were log_10_ (x + 1) transformed and subjected to the Shapiro-Wilk test to determine the statistical assumption of normally distributed data. The catch per unit effort (CPUE; individual/hour) was calculated as the total number of fish individuals/(sampling effort × 12 h). Based on normally distributed data (*p* > 0.05), Student’s t-test was conducted to compare the CPUE of native fish species between near-cage and far-cage sites using IBM SPSS software version 21 (Arbuckle, 2012; Coakes, 2013). The introduced species were excluded from the analysis. The status of fish species, either native or introduced, was categorised based on Chong, Lee & Lau (2010), Kottelat (2013), and Rahim, Esa & Arshad (2013).

**Species richness–**Data for species richness was first standardised by the rarefaction method. Rarefaction is a statistical procedure to standardize data due to unequal sample numbers among sites (Hughes et al., 2001). Thus, a valid comparison of species richness from samples of different sizes can be made after conducting “rarefaction” to standardize the number of individuals among sites (Chiarucci et al., 2009). The rarefaction procedure was created by scaling down the number of individuals to the lowest number incorporated in the EcoSim statistical software version 7 (Gotelli & Entsminger, 2001). The parametric analysis was performed on normally distributed data (*p* > 0.05). A Student’s t-test was conducted to determine the species richness of native fish species between near-cage and far-cage sites using IBM SPSS software version 21 (Arbuckle, 2012).

**Principal component analysis (PCA)–**The species were categorised based on their diet (feeding habit) and habitat. Information on these biological traits was referred to from various sources, including Rainboth (1996), Mazlan et al. (2007), Ambak et al. (2010), Shaharom (2012), Mustafa-Kamal et al. (2012), and FishBase (Froese & Pauly, 2016) and was coded accordingly (Supplemental Material). For each trait, each species was scored as 1 for presence and 0 for absence. The overlapping ecological requirements among these fish species according to diet and habitat were determined. Principal component analysis (PCA) was performed by PAST software version 2.1 (Hammer, Harper & Ryan, 2001) using data compiled from the biological traits of all the species. A biplot was generated using the eigenvalue scale of component 1 and component 2.

## Results

### Fish assemblages

A total of nine families comprising 16 fish species were recorded during this study. Cyprinidae was the most dominant family with six species, while the other families were only represented by one or two species (Table 1). Fifteen species were recorded at the near-cage site, with nine species classified as “less frequent” while 16 species were recorded at the far-cage site, with 11 species classified as “less frequent”. Observed fish species number at the far-cage site, therefore, surpassed that at the near-cage site by only one species, *Anabas testudineus*.

**Table 1:** Fish species checklist recorded at near-cage and far-cage sites from the AIZ of Temengor Reservoir, Perak.

Family	Species	Common name	Category	Near-cage	Far-cage
**Anabantidae**	*Anabas testudineus*	Climbing perch	Native	–	*_LF_
**Bagridae**	*Hemibagrus nemurus*	Asian redtail catfish	Native	*_LF_	+_LF_
**Channidae**	*Channa micropeltes*	Giant snakehead	Native	+	+_LF_
	*Channa striata*	Striped snakehead	Native	*_LF_	+_LF_
**Cichlidae**	*Oreochromis niloticus*	Tilapia	Introduced	+	+_LF_
**Cyprinidae**	*Cyclocheilichthys apogon*	Beardless barb	Native	+	+
	*Hampala macrolepidota*	Hampala barb	Native	+	+
	*Labiobarbus fasciatus*	Barb	Native	+_LF_	+_LF_
	*Mystacoleucus obtusirostris*	Minnow	Native	+_LF_	+
	*Osteochilus vittatus*	Bonylip barb	Native	+	+
	*Oxygaster anomalura*	Glassfish	Native	+	+
**Eleotridae**	*Oxyeleotris marmorata*	Marble goby	Native	+_LF_	+_LF_
**Notopteridae**	*Notopterus notopterus*	Bronze featherback	Native	+_LF_	+_LF_
**Osphronemidae**	*Osphronemus goramy*	Giant gourami	Native	+_LF_	+_LF_
	*Trichogaster pectoralis*	Snakeskin gourami	Introduced	+_LF_	+_LF_
**Pristolepididae**	*Pristolepis fasciata*	Malayan leaffish	Native	+	+_LF_
	**Total number of individuals**			585	1,048
	**Total number of species**			15	16
	**Total number of less frequent species caught**			9	11
	**Total number of families**			8	9
	**Sampling effort**			15	9
	**CPUE (ind/hour)**			3.250	9.703

**Note:**

‘−’, absent; ‘*’, recorded ≤2 individuals; ‘+’, recorded >2 individuals; ‘LF’, less frequent species caught (total individuals of species caught <2% of the total catch). The bold text indicates the different indices.

Two introduced species were recorded, the snakeskin gourami, *Trichogaster pectoralis* (Osphronemidae), and tilapia, *Oreochromis niloticus* (Cichlidae), while the other species were native. In particular, the near-cage site recorded 562 native fish specimens, whereas the far-cage site recorded 1,032 native fish specimens, represented by 13 and 14 fish species, respectively (Table 2). Both introduced species were recorded at the two sites. Of the 28 *O. niloticus* specimens caught, 20 were at the near-cage site, and eight individuals were at the far-cage site (Table 2). Based on Student’s t-test, the CPUE (individual/hour) of native fish species at the far-cage site of Temengor Reservoir was found to be significantly higher (*p* < 0.05) than that at the near-cage site (Supplemental Material).

**Table 2:** Total number of individuals, species, families of native and tilapia species, sampling effort, and CPUE recorded at near-cage and far-cage sites from the AIZ of Temengor Reservoir, Perak.

	Native fish		Tilapia	
	Near-cage	Far-cage	Near-cage	Far-cage
Total number of individuals	562	1,032	20	8
Total number of species	13	14	1	1
Total number of families	7	8	–	–
Sampling effort	15	9	15	9
CPUE (ind/hour)	3.122	9.556	0.112	0.074

**Figure 3:**
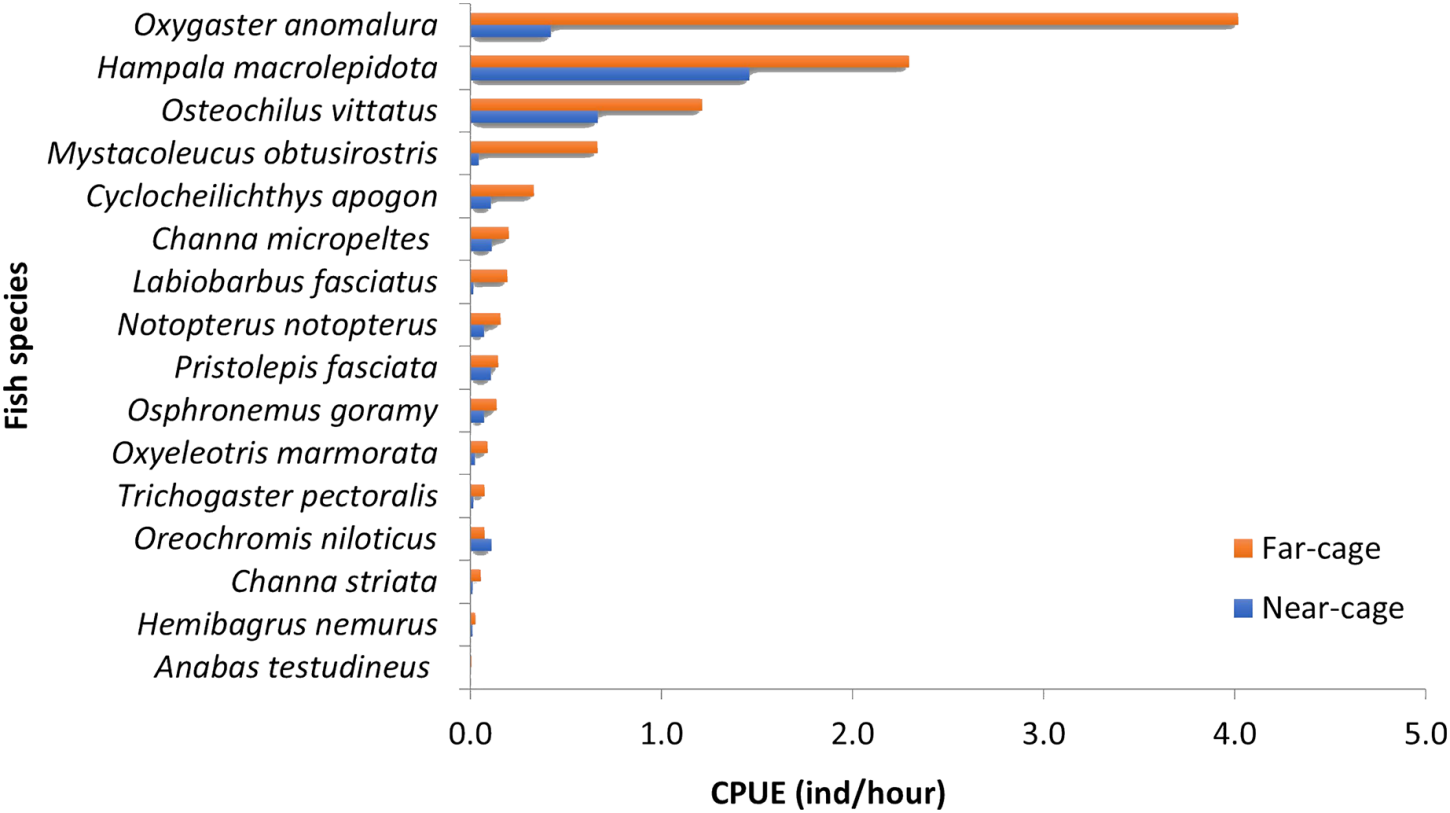
Catch per unit effort (CPUE) for each species caught at near-cage and far-cage sites from the AIZ of Temengor Reservoir during the study period.

The absolute values for each species’ CPUE (ind/hour) were different between near-cage and far-cage sites (Fig. 3), although the dominance patterns were similar (except for *O. niloticus*). *Oxygaster anomalura, Hampala macrolepidota,* and *Osteochilus vittatus* were the three species that dominated the catches during this study. At the near-cage site, the CPUE of *H. macrolepidota* was 1.46, followed by *O. vittatus* and *O. anomalura*, which had values of 0.67 and 0.42, respectively. At the far-cage site, *O. anomalura* represented the highest catch with CPUE of 4.02, followed by *H. macrolepidota* and *O. vittatus*, which had values of 2.30 and 1.21, respectively. Although not significant (*p* > 0.05), it was apparent that the tilapia, *O. niloticus*, showed higher CPUE at near-cage compared to the far-cage site. In contrast, all the other species showed higher CPUEs at the far-cage compared to the near-cage site. In particular, *Oxygaster anomalura, Mystacoleucus obtusirostris* and *Labiobarbus fasciatus* recorded >50% CPUE than that at the near-cage site.

### Species richness of native fish assemblages

**Figure 4:**
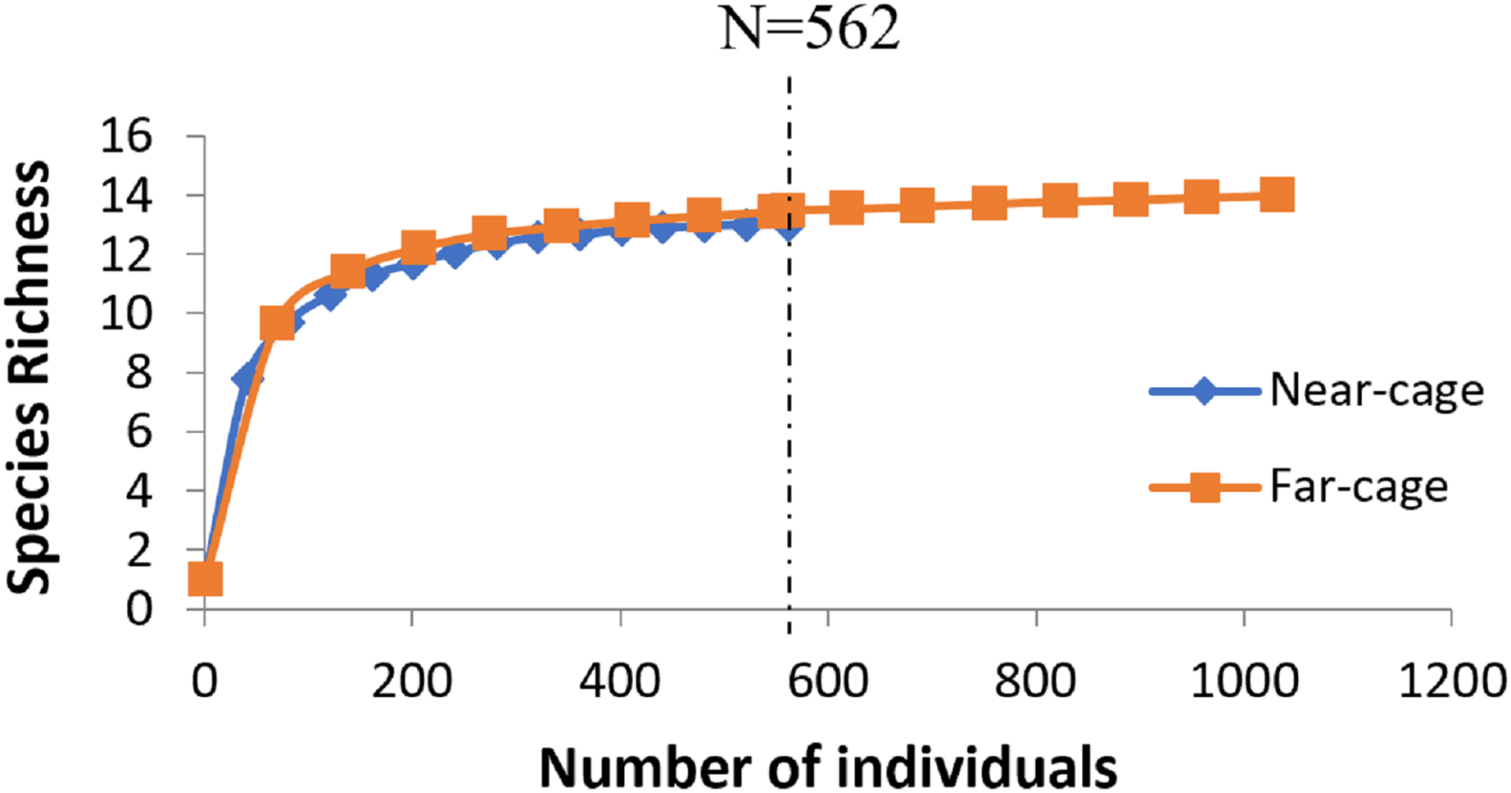
Rarefaction curve of species richness of native fish assemblages at near-cage and far-cage sites from the AIZ of Temengor Reservoir conducted at *N* = 562.

The average rarefaction for species richness (Fig. 4) based on the lower value (562) recorded at the near-cage site shows that the index was slightly higher at the far-cage (13.485) compared to that at the near-cage site (13.000), although with not significantly different (*p* > 0.05).

### Principal component analysis (PCA) based on the diet and habitat preferences

**Figure 5:**
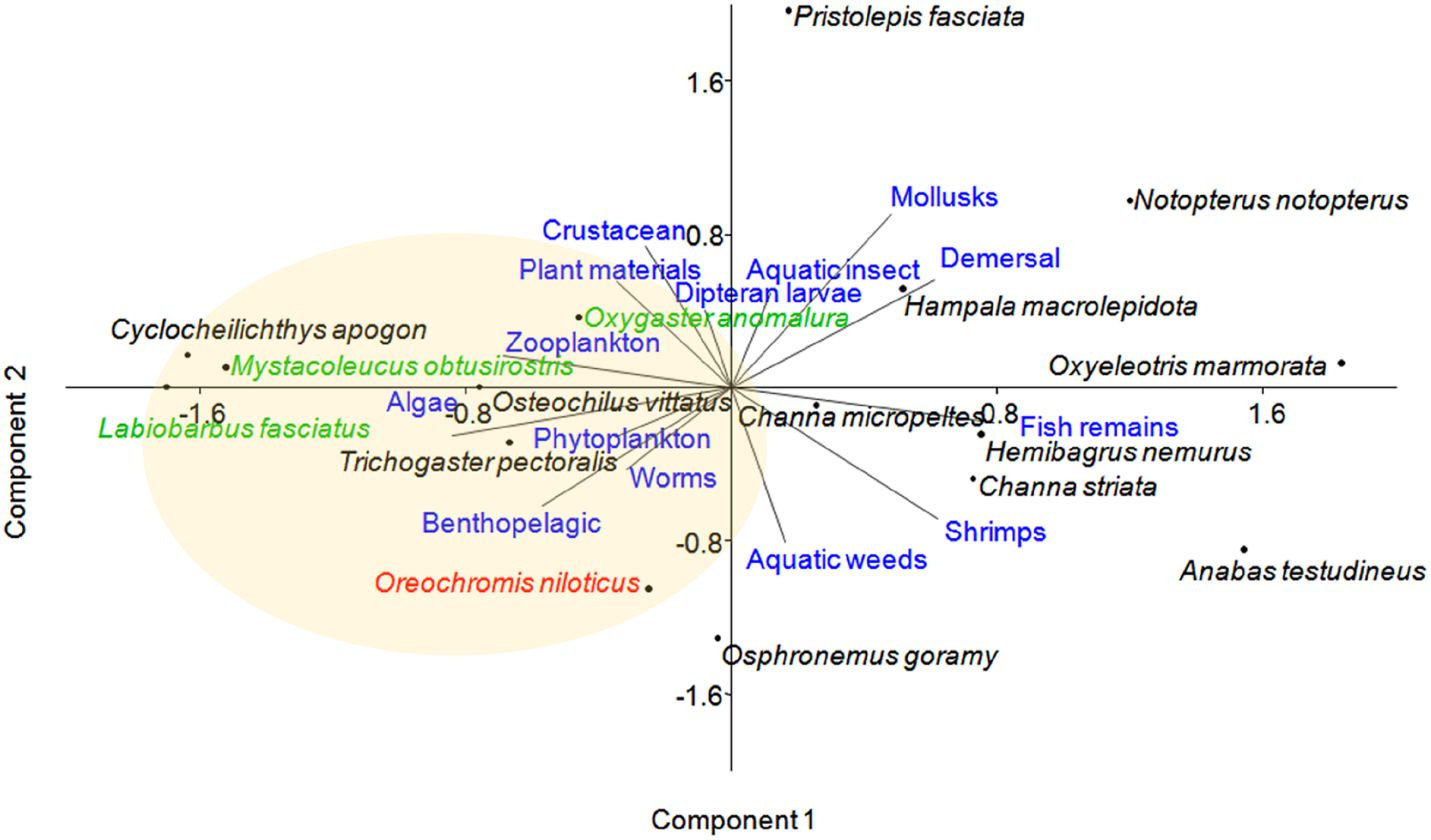
Distribution of fish species with respect to diet and habitat variables, identified by principal component analysis (PCA). Several traits were omitted from the figure for clarity. Fish species in green show >50% less CPUE at the near-cage site than that at the far-cage site.

Principal component analysis (PCA) of 16 fish species observed at near-cage and far-cage sites during this study was grouped based on the diet and habitat preferences (Fig. 5; Supplemental Material). The first two principal components (PC1 and PC2) of fish assemblage ordination explained 44.72% of the variation (Table 3). The analysis showed that the tilapia, *O. niloticus* had almost overlapping diet resources and habitat with other native fish species. Tilapia diets are phytoplankton, algae, plant materials, aquatic weeds, and fish remains. The phytoplankton and algae diets are also in common with *L. fasciatus, M. obtusirostris, C. apogon, O. vittatus*, and *T. pectoralis*. In addition, the plant materials are shared by *C. apogon, O. vittatus, Hemibagrus nemurus, L. fasciatus, O. anomalura, Notopterus notopterus*, and *Pristolepis fasciata*, whereas aquatic weeds are common to *O. goramy* and *A. testudineus*. The fish remains diet is shared by *A. testudineus, Channa micropeltes, C. striata, Hampala macrolepidota, H. nemurus, N. notopterus, Oxyeleotris marmorata*, and *O. anomalura*. Based on these similar ecological requirements, *O. niloticus* may outcompete native species, especially *O. anomalura*, *L. fasciatus*, and *M. obtusirostris*, which were found to be in low numbers at the near-cage site (Fig. 3).

**Table 3:** Eigenvalues, variances, and cumulative percentages based on principal component analysis (PCA) for habitat and trophic characteristics of fish species at near-cage and far-cage sites from the AIZ of Temengor Reservoir, Perak.

PCs	Eigenvalue	Variance (%)	Cumulative of variance (%)
1	1.184	29.52	29.52
2	0.610	15.21	44.72
3	0.576	14.36	59.08
4	0.461	11.48	70.56

## Discussion

Out of 16 fish species observed in this study, two introduced species were recorded at both near-cage and far-cage sites from the AIZ of Temengor Reservoir, namely *Trichogaster pectoralis* and *Oreochromis niloticus*. Since their introduction into the aquaculture industry more than 50 years ago, escapees have successfully established feral populations in the local habitats of Malaysia. Chong, Lee & Lau (2010) reported that *T. pectoralis* has not threatened the native fish species and thus, is not considered invasive. On the other hand, *O. niloticus* can be considered invasive as their numbers rapidly increase and often displaces native species, particularly in lakes and ponds such as at Lake Victoria, East Africa (Zengeya et al., 2013), Pokhara Valley, Nepal (Husen, 2014), Pearl River and Jianjiang River, South China (Gu et al., 2014), Hombolo Lake, Tanzania (Turner, Ngatunga & Genner, 2019) and Lake Kutubu, Papua New Guinea (Thresher, Smith & Cutajar, 2020). The relatively higher tolerance of tilapia to habitat degradation, even juvenile tilapia evidently adapted to polluted areas of Paraíba do Sul River, Brazil, could be regarded as a primary cause of species invasiveness (Linde et al., 2008).

This present study validates earlier reports of tilapia escapees from the fish cage at the AIZ. According to Shah et al. (2016a), there were no sightings of *O. niloticus* at Temengor Reservoir between 1973–2000. Similarly, a fish checklist by Hashim et al. (2012) did not record the presence of tilapia between 2001–2002. An assessment of fish community distribution by Zainudin (2005) did not record any tilapia presence. Moreover, Ibrahim (2016) did not observe any tilapia individuals in 2008. These earlier studies provide strong evidence on the absence of tilapia at Temengor Reservoir before the establishment of tilapia cage culture in 2008. However, a total of 28 tilapia specimens were caught in this study; 20 were found at the near-cage site, while another eight individuals were caught at the far-cage site. These are sizeable numbers and are a worrying trend as it proves that the tilapia is escaping from their ‘secure’ cages.

Incidences of escaped cage-cultured fish are prevalent. Although aquaculture involves fish culture in confined areas such as ponds, pens, or tanks, the risks of accidental releases into natural waters still exist (Naylor et al., 2004; Arthur et al., 2010). A review by Senanan & Bart (2010) on the risk of escaped tilapia proposed that the pattern of escape varied from regular escapes of low numbers of escapees (during production cycles) to less frequent escapes of high numbers of escapees per event (during a natural catastrophe or harsh weather conditions such as a storm and flood). These escapes could occur due to any damages to the cage net by human errors or predators, causing holes and breakdown of the cage (Anderson, 2004). The escape of tilapia from the cages at Temengor Reservoir could be attributed to one or more of these factors.

Three species dominated both the near-cage and far-cage sites; *Oxygaster anomalura, Hampala macrolepidota*, and *Osteochilus vittatus*, demonstrating that only a handful of species contribute to total abundance in the lake. The catch per unit effort (CPUE) of native fish specimens at the far-cage site was significantly higher than that at the near-cage site, showing that the presence of escaped tilapia substantially influences the abundance of native fish at Temengor Reservoir. The trend of decreased CPUE has also been highlighted by Gu et al. (2015), where the CPUE of native fish species in the main rivers of Guangdong Province, China had a significant negative correlation with the abundance of Nile tilapia.

The rarefaction analyses showed that the far-cage site harbored a slightly higher richness of native fish composition (although not significant) than that at near-cage site when an equal number of individuals were compared. The index indicated that the native fish numbers are dwindling (*e.g*., near-cage site) when the escapees are abundant. Previous study reported on the decline of native fish as the impact of the introduction of invasive *O. niloticus*. Similarly, in a study by Gu et al. (2014), native fish species richness showed a significant negative correlation with both the weight ratio and the total catch of Nile tilapia. According to Husen (2014), there was increasing trends of Nile tilapia and decreasing trends of native fish species catches from lakes of Pokhara Valley. Thresher, Smith & Cutajar (2020) reported the tilapia invasion as the cause of the decline in native fishes in Lake Kutubu, Papua New Guinea. In a worst-case scenario, the colonization of *O. niloticus* coincided with the extinction of native cyprinids in Lake Lanao, Mindanao, whereas in north-east Thailand, the tilapia replaced native species and became pests in open waters after excessive reproduction success at public waters (natural lakes and man-made reservoirs) (Stauffer et al., 2022). Turner, Ngatunga & Genner (2019) proposed that *O. niloticus* incursions led to the local extinction of the native tilapia species *O. urolepis* from Hombolo Lake, Tanzania. Based on the reported evidence of native species loss due to the tilapia invasion, fortunately, the observed similar levels of fish species richness between near-cage and far-cage sites from the AIZ of Temengor Reservoir indicate that the presence of tilapia fish cages has not yet resulted in the extinction of native species.

The lower number of native fish catch at the near-cage site could be explained by competition with tilapia escapees with similar ecological requirements. Several studies have reported the reduced catches of native species in a similar scenario, attributed to predation by tilapia due to habitat and trophic overlaps (McKaye et al., 1995; Peterson et al., 2004; Peterson, Slack & Woodley, 2005; Canonico et al., 2005; Thresher, Smith & Cutajar, 2020; Stauffer et al., 2022). Arthur et al. (2010) reported that the Nile tilapia had reduced the native fish communities in southern Lao PDR. The Nile tilapia *O. niloticus* is known to utilize phytoplankton and blue-green algae that are important food sources to native species in the region. The competition between tilapia and native species for food and breeding territories, posing a threat to fish populations in the Darling River system, Australia (Bryceson-Noragric, 2005). In the Jatigede Reservoir, Indonesia, *O. niloticus* has moderate value of overlapping food with native fish including the three spotted gourami *Trichopodus trichopterus*, common barb *Mystacoleucus marginatus*, and bonylip barb *Osteochilus vittatus*. Subsequently, the intense competition in food has negative effects on native species (Herawati et al., 2020; Radkhah & Eagderi, 2021). Champneys, Genner & Ioannou (2021) stated that *O. niloticus* threaten native species by dominating interference competition. Based on these evidence, we believe the overlapping diet requirements with the tilapia could be attributed to the reduction of several native species abundance at the near-cage site of Temengor Reservoir.

The PCA analysis placed several Cyprinids such as *O. anomalura*, *L. fasciatus, Mystacoleucus obtusirostris, Cyclocheilichthys apogon* and *O. vittatus*, in the same group as tilapia, which highlights potential competition among these species due to common feeding habit and habitat requirements. The CPUE analysis of native species strongly indicated that this is already occurring at Temengor Reservoir where tilapia, a well recognised voracious feeder (Beveridge et al., 1988; Yusufzai et al., 2020), is outcompeting these native species. Hence, three species (*O. anomalura*, *L. fasciatus, M. obtusirostris*) appeared to be most affected by the aggressive competition from tilapia, evident by the >50% reduction of CPUE at the near-cage site as compared to the far-cage site. Tilapia is hardy and adaptable in varied types of habitat and, therefore has higher survival ability. In the long term, other native fish are predicted to suffer the same fate at the Temengor Reservoir. In addition, tilapia escapees may compete with native species for breeding sites, spawning grounds, and nurseries, thereby reducing their abundance not only through limited food resources but also inhibiting their growth and reproductive success (Njiru et al., 2005; Senanan & Bart, 2010; Beatty & Morgan, 2013; Zengeya et al., 2013). If left unmanaged, the situation could deteriorate with adverse consequences on the lake’s biodiversity.

The success of *O. niloticus* as an invasive species has been attributed to its opportunistic feeding behavior (Getabu, 1994; Njiru et al., 2004). Despite originating from a protected environment in an aquaculture facility, escapees appeared adaptable to a broad range of diets (Zengeya et al., 2013). Members of the cichlid family have evolved adaptations to eating every conceivable food source in their environment. They have both mouth and throat jaws that can speed up the breakdown of food. This allows them to consume food, ranging from algae to scales of other fish, with no limitation to any particular food sources (Meyer, 2015). Their broad range diet helps to alleviate some of the competition with the native fish species at the near-cage site of Temengor Reservoir.

Perhaps, even more detrimental to other co-habiting species is the tilapia’s carnivorous feeding habit of predating on the eggs, fry and small fish of many higher trophic level species (Canonico et al., 2005; Vicente & Fonseca-Alves, 2013; Gu et al., 2015; Thresher, Smith & Cutajar, 2020). As highlighted by Martin, Valentine & Valentine (2010), the impacts of adult tilapia on native ecosystems and food webs could be further exacerbated by the fact that they may be more competitive with larger consumers. A study by de Moor, Wilkinson & Herbst (1986) on the feeding habit of tilapia at Hartbeespoort Dam, South Africa, proved that tilapias are known to feed on smaller fish and fish eggs. In another study at Virgin River, USA, tilapias were believed to prey on, or compete with, other native fish such as the endangered woundfin *Plagopterus argentissimus*, and chub *Gila seminude*. A stomach content analysis indicated that they are omnivorous, feeding on a range of vegetable and animal material, including fish (Canonico et al., 2005). Based on these examples, the introduced tilapia at Temengor Reservoir could well predate eggs and fries of native fish, even though reductions are still minimal at the near-cage site.

The current study could be considered the first detailed investigation of this issue at the Temengor Reservoir. Still, more data is needed for a comprehensive management and conservation program of this important ecosystem. Undoubtedly, the positive impact of established tilapia populations as important sources of food and income to local people, such as in Papua New Guinea and Sri Lanka (de Silva et al., 2004), is well acknowledged. However, the negative impacts on the ecosystem, as described above, could far outweigh the positive impact if not properly managed, especially in a large water body and connecting rivers. In the context of Temengor Reservoir, *O. niloticus* has been recorded at Kejar River, approximately 40 km from the AIZ (Ibrahim, 2016; Abd Hamid et al., 2022b). This suggested that the escaped tilapia could potentially colonise the lake and connecting rivers. Several studies have shown that they could efficiently disperse from the point of introduction to connecting water bodies within the catchment area (Thresher, Smith & Cutajar, 2020; Stauffer et al., 2022). For instance: (i) *Oreochromis niloticus* was first introduced for aquaculture in the Kafue River watershed in Zambia in 1982, and by the middle of the 1990s, escapees had been detected in the river; (ii) In the early 2000s, tilapia was farmed on Lake Kariba in Zambia, and the escapees have since spread to most of the middle Zambezi River and other drainages.

The impacts of escapees on the native fish species may only become fully apparent years or decades after the first introduction (Strayer et al., 2006; Spens, Englund & Lundqvist, 2007; Arthur et al., 2010). To date, native fish species diversity at Temengor Reservoir is still maintained at a healthy level. Presumably the number of escapees is still at a very low level and thus has not led to any significant impacts on native fish diversity. Moreover, the aquaculture facility at AIZ has only been established in the last seven years. Generally, the introduction of non-native species could affect the stability of ecosystems, leading to native extinction through long term predation and competition (Kour, Bhatia & Sharma, 2014). In Lake Luhondo, Rwanda, the incursion of *O. niloticus* corresponded with the complete disappearance of the large cyprinids, *Barbus microbarbis* and *Varicorhinus ruandae* in 1952, just 14–17 years after the introduction (De Vos, Snoeks & van den Audenaerde, 1990). However, while the timeframe of the exposure in the present study might be insufficient to manifest the full effects of the invasive tilapia on the native fish fauna, a trend of reduced species populations is already evident. The native fish species may face tremendous risk and reduction if the situation continues for a longer time in the next 10 or 20 years. Therefore, immediate actions need to be taken to control the presence and spread of tilapia in the natural waters. Previous studies have described several approaches, including chemical (rotenone and antimycin), physical (casting nets, gill nets, traps, and electrofishing), and biological (biotic resistance and biological control by native carnivorous fish) to address this issue. However, each method has its pros and cons (Knapp & Matthews, 1998; Britton & Brazier, 2006; Sato et al., 2010), and we hope through a more holistic study, the optimal approach(es) could be defined and implemented.

## Conclusions

This study showed a correlative trend between the presence of tilapia in the natural waters of Temengor Reservoir and the lower number of native fish catch. The PCA analysis revealed that the tilapia may have a competitive advantage over the members of its “habitat and trophic” group, highlighted by the slight decrease of certain native fish abundance particularly *Oxygaster anomalura*, *Labiobarbus fasciatus*, and *Mystacoleucus obtusirostris*. Considering the adaptable feeding behavior of tilapia, the native fish populations are susceptible to the risk of decline due to long-term competition and predation by escaped tilapia. The expansion of aquaculture facilities for this species may pose a threat due to its invasive condition.

## Supplemental Information

10.7717/peerj.15986/supp-1 Supplemental Information 1 Raw data count of fish assemblages at near-cage and far-cage sites of Temengor Reservoir, Perak.

10.7717/peerj.15986/supp-2 Supplemental Information 2 Script and statistical analyses for the CPUE (individual/hour) of native fish species at both near-cage and far-cage sites of Temengor Reservoir, Perak.

10.7717/peerj.15986/supp-3 Supplemental Information 3 Script and statistical analyses for the ecological indices of native fish at both near-cage and far-cage sites of Temengor Reservoir, Perak.

10.7717/peerj.15986/supp-4 Supplemental Information 4 Feeding habit (diet) and habitat characteristics of species caught at near-cage and far-cage sites from AIZ, Temengor Reservoir. Ates = *Anabas testudineus*, Cmic = *Channa micropeltes*, Cstr = *Channa striata*, Capo = *Cyclocheilichthys apogon*, Hmac = *Hampala macrolepidota*, Hnem = *Hemibagrus nemurus*, Lfas = *Labiobarbus fasciatus*, Mobt = *Mystacoleucus obtusirostris*, Nnot = *Notopterus notopterus*, Onil = *Oreochromis niloticus*, Ogor = *Osphronemus goramy*, Ovit = *Osteochilus vittatus*, Omar = *Oxyeleotris marmorata*, Oano = *Oxygaster anomalura*, Tpec = *Trichogaster pectoralis*, Pfas = *Pristolepis fasciata*, 1 = Yes, 2 = No

## Additional Information and Declarations

### Data Availability

The following information was supplied regarding data availability: The raw data is available in the Supplemental Files.
