# Supplementary material for: Impacts of tilapia aquaculture on native fish diversity at an ecologically important reservoir
Source: PeerJ. 2023 Dec 19;11:e15986. doi: 10.7717/peerj.15986 (PMC10740594; doi:10.7717/peerj.15986)
Supplement: Supplemental Information 2 [file peerj-11-15986-s002.pdf]

File 2: Script and statistical analyses for the CPUE (individual/hour) of native fish species at both near-cage and far-cage sites of Temengor Reservoir, Perak

Site	SPSS	Cpue-effort
Near-cage	1	1.833
Near-cage	1	2.042
Near-cage	1	1.208
Near-cage	1	3.750
Near-cage	1	2.458
Near-cage	1	1.167
Near-cage	1	0.375
Near-cage	1	0.250
Near-cage	1	0.917
Near-cage	1	1.583
Near-cage	1	2.000
Near-cage	1	1.542
Near-cage	1	0.792
Near-cage	1	0.583
Near-cage	1	2.917
Far-cage	2	3.292
Far-cage	2	4.333
Far-cage	2	3.375
Far-cage	2	4.292
Far-cage	2	4.958
Far-cage	2	6.292
Far-cage	2	7.458
Far-cage	2	3.292
Far-cage	2	5.708

Tests of Normality

	Kolmogorov-Smirnov[a]			Shapiro-Wilk		
	Statistic	df	Sig.	Statistic	df	Sig.
CPUE	.144	24	.200*	.936	24	.130

*. This is a lower bound of the true significance.

a. Lilliefors Significance Correction

Independent Samples Test

		Levene's Test for Equality of Variances		t-test for Equality of Means						
		F	Sig.	t	df	Sig. (2-tailed)	Mean Difference	Std. Error Difference	95% Confidence Interval of the Difference	
									Lower	Upper
CPUE	Equal variances assumed	2.363	.138	-6.480	22	.000	-3.216667	.496420	-4.246179	-2.187155
	Equal variances not assumed			-5.852	12.312	.000	-3.216667	.549684	-4.410972	-2.022361
