# Supplementary material for: Impacts of tilapia aquaculture on native fish diversity at an ecologically important reservoir
Source: PeerJ. 2023 Dec 19;11:e15986. doi: 10.7717/peerj.15986 (PMC10740594; doi:10.7717/peerj.15986)
Supplement: Supplemental Information 3 [file peerj-11-15986-s003.pdf]

File 3: Script and statistical analyses for the ecological indices of native fish at both near-cage and far-cage sites of Temengor Reservoir, Perak

Site	Code	Richness	Shannon	Hulbert's	Dominance
Near-cage	1	4.9010	1.4405	0.8202	0.3676
Near-cage	1	4.4590	1.2731	0.7432	0.4791
Near-cage	1	3.3770	1.0159	0.6611	0.5278
Near-cage	1	4.3840	1.3252	0.7688	0.4344
Near-cage	1	3.3680	0.9628	0.5861	0.6144
Near-cage	1	2.8350	0.8217	0.5637	0.6169
Near-cage	1	4.0000	1.2149	0.7500	0.4444
Near-cage	1	2.0000	0.6365	0.5000	0.6667
Near-cage	1	3.6070	1.1049	0.7035	0.4952
Near-cage	1	2.5350	0.6450	0.4417	0.7359
Near-cage	1	3.7700	1.1301	0.6941	0.5057
Near-cage	1	4.0940	1.2756	0.7657	0.4243
Near-cage	1	1.9340	0.3239	0.2122	0.8926
Near-cage	1	2.5380	0.6044	0.3843	0.7811
Near-cage	1	2.6940	0.7283	0.4915	0.6831
Far-cage	2	3.0290	0.8228	0.5211	0.6840
Far-cage	2	3.3580	0.8919	0.5606	0.6464
Far-cage	2	4.1860	1.2203	0.7270	0.4751
Far-cage	2	4.4570	1.2898	0.7503	0.4672
Far-cage	2	4.9360	1.3977	0.7788	0.4442
Far-cage	2	3.4360	1.0641	0.6741	0.5183
Far-cage	2	4.7910	1.4047	0.7934	0.4097
Far-cage	2	2.9820	0.8629	0.5691	0.6140
Far-cage	2	2.2500	0.6099	0.4443	0.7217

Tests of Normality

		Kolmogorov-Smirnov[a]			Shapiro-Wilk		
		Statistic	df	Sig.	Statistic	df	Sig.
Richness	1	.119	15	.200*	.962	15	.723
	2	.174	9	.200*	.944	9	.621
Shannon	1	.131	15	.200*	.952	15	.561
	2	.172	9	.200*	.931	9	.488
Hulbert's	1	.163	15	.200*	.924	15	.220
	2	.183	9	.200*	.919	9	.381
Dominance	1	.164	15	.200*	.950	15	.518
	2	.197	9	.200*	.916	9	.356

*. This is a lower bound of the true significance.

a. Lilliefors Significance Correction

Independent Samples Test

		Levene's Test for Equality of Variances		t-test for Equality of Means						
		F	Sig.	t	df	Sig. (2-tailed)	Mean Difference	Std. Error Difference	95% Confidence Interval of the Difference	
									Lower	Upper
Richness	Equal variances assumed	.019	.893	-.898	22	.379	-.3474889	.3871391	-1.1503662	.4553884
	Equal variances not assumed			-.897	16.928	.383	-.3474889	.3875628	-1.1654409	.4704631
Shannon	Equal variances assumed	.325	.575	-.731	22	.472	-.0958244	.1310180	-.3675392	.1758903
	Equal variances not assumed			-.759	18.979	.457	-.0958244	.1262217	-.3600292	.1683803
Hulbert's	Equal variances assumed	.954	.339	-.616	22	.544	-.0407822	.0662158	-.1781055	.0965410
	Equal variances not assumed			-.667	20.947	.512	-.0407822	.0611849	-.1680426	.0864782
Dominance	Equal variances assumed	.857	.365	.420	22	.679	.0245467	.0584461	-.0966631	.1457565
	Equal variances not assumed			.450	20.558	.657	.0245467	.0545155	-.0889731	.1380665
