# Supplementary material for: Impacts of tilapia aquaculture on native fish diversity at an ecologically important reservoir
Source: PeerJ. 2023 Dec 19;11:e15986. doi: 10.7717/peerj.15986 (PMC10740594; doi:10.7717/peerj.15986)
Supplement: Supplemental Information 4 — Ates = Anabas testudineus, Cmic = Channa micropeltes, Cstr = Channa striata, Capo = Cyclocheilichthys apogon, Hmac = Hampala macrolepidota, Hnem = Hemibagrus nemurus, Lfas = Labiobarbus fasciatus, Mobt = Mystacoleucus obtusirostris, Nnot = Notopterus notopterus, Onil = Oreochromis niloticus, Ogor = Osphronemus goramy, Ovit = Osteochilus vittatus, Omar = Oxyeleotris marmorata, Oano = Oxygaster anomalura, Tpec = Trichogaster pectoralis, Pfas = Pristolepis fasciata, 1 = Yes, 2 = No [file peerj-11-15986-s004.pdf]

File 4: Feeding habit (diet) and habitat characteristics of species caught at near-cage and far-cage sites from AIZ, Temengor Reservoir. *Ates* = *Anabas testudineus, Cmic* = *Channa micropeltes, Cstr* = *Channa striata, Capo* = *Cyclocheilichthys apogon, Hmac* = *Hampala macrolepidota, Hnem* = *Hemibagrus nemurus, Lfas* = *Labiobarbus fasciatus, Mobt* = *Mystacoleucus obtusirostris, Nnot* = *Notopterus notopterus, Onil* = *Oreochromis niloticus, Ogor* = *Osphronemus goramy, Ovit* = *Osteochilus vittatus, Omar* = *Oxyeleotris marmorata, Oano* = *Oxygaster anomalura, Tpec* = *Trichogaster pectoralis, Pfas* = *Pristolepis fasciata*, 1 = Yes, 2 = No

**Traits**		**Fish species**															
		Ates	*Cmic*	*Cstr*	*Capo*	*Hmac*	*Hnem*	*Lfas*	*Mobt*	*Nnot*	***Onil***	*Ogor*	*Ovit*	*Omar*	*Oano*	*Tpec*	*Pfas*
**Diet**	Plant materials	0	0	0	1	0	1	1	0	1	**1**	0	1	0	1	0	1
	Aquatic weeds	1	0	0	0	0	0	0	0	0	**1**	1	0	0	0	0	0
	Algae	0	0	0	1	0	0	1	1	0	**1**	0	1	0	0	1	0
	Filamentous algae	0	0	0	1	0	0	1	1	0	**1**	1	0	0	1	1	1
	Phytoplankton	0	0	0	0	0	0	1	0	0	**1**	0	1	0	0	0	0
	Zooplankton	0	0	0	1	0	0	1	1	0	**0**	0	0	0	1	0	0
	Aquatic insect	0	0	1	1	1	1	0	1	1	**0**	1	0	1	1	1	1
	Dipteran larvae	0	0	1	0	0	0	0	1	0	**0**	0	0	0	0	0	1
	Worms	0	0	1	1	0	0	0	1	0	**0**	1	0	0	0	0	0
	Crustacean	0	1	1	1	1	1	1	1	1	**0**	0	1	0	0	0	1
	Mollusks	0	0	0	0	1	0	0	0	1	**0**	0	0	1	0	0	1
	Fish remains	1	1	1	0	1	1	0	0	1	**1**	0	0	1	1	0	0
	Shrimps	1	0	1	0	0	1	0	0	0	**0**	1	0	1	0	0	0
	Crabs	0	0	0	0	0	0	0	0	0	**0**	0	0	1	0	0	0
	Fruits	0	0	0	0	0	0	0	0	0	**0**	0	0	0	0	0	1
	Seeds	0	0	0	0	0	0	0	0	0	**0**	0	0	0	0	0	1
**Habitat**	Pelagic	0	0	0	0	0	0	0	0	0	**0**	0	0	0	1	0	0
	Benthopelagic	0	1	1	1	1	1	1	1	0	**1**	1	1	0	0	1	0
	Demersal	1	0	0	0	0	0	0	0	1	**0**	0	0	1	0	0	1
	Brackish	1	0	1	0	0	1	0	0	1	**1**	1	0	1	0	0	0
	Potamodromus	1	1	1	0	1	1	0	0	1	**1**	0	1	1	0	1	1
